# The Study of Structural, Impedance and Energy Storage Behavior of Plasticized PVA:MC Based Proton Conducting Polymer Blend Electrolytes

**DOI:** 10.3390/ma13215030

**Published:** 2020-11-07

**Authors:** Shujahadeen B. Aziz, Iver Brevik, M. A. Brza, A. S. F. M. Asnawi, Elham M. A. Dannoun, Y. M. Yusof, Rebar T. Abdulwahid, M. H. Hamsan, Muaffaq M. Nofal, M. F. Z. Kadir

**Affiliations:** 1 Hameed majid Advanced Polymeric Materials Research Lab., Physics, College of Science, University of Sulaimani, Qlyasan Street, Kurdistan Regional Government-Iraq, Sulaimani 46001, Iraq; mohamad.brza@gmail.com (M.A.B.); rebar.abdulwahid@univsul.edu.iq (R.T.A.); 2 Department of Civil engineering, College of Engineering, Komar University of Science and Technology, Kurdistan Regional Government, Sulaimani 46001, Iraq; 3 Department of Energy and Process Engineering, Norwegian University of Science and Technology, N-7491 Trondheim, Norway; 4 Department of Manufacturing and Materials Engineering, Faculty of Engineering, International Islamic University of Malaysia, Kuala Lumpur 53100, Malaysia; 5 Chemical Engineering Section, Universiti Kuala Lumpur, Malaysian Institute of Chemical & Bioengineering Technology (UniKL MICET), Alor Gajah 78000, Malacca, Malaysia; asyafiq.asnawi@s.unikl.edu.my (A.S.F.M.A.); yuhanees@unikl.edu.my (Y.M.Y.); 6 Associate Director of General Science Department, Woman Campus, Prince Sultan University, P. O. Box 66833, Riyadh 11586, Saudi Arabia; elhamdannoun1977@gmail.com; 7 Department of Physics, College of Education, University of Sulaimani, Old Campus, Kurdistan Regional Government, Sulaimani 46001, Iraq; 8 Institute for Advanced Studies, University of Malaya, Kuala Lumpur 50603, Malaysia; hafizhamsan93@gmail.com; 9 Department of Mathematics and General Sciences, Prince Sultan University, P. O. Box 66833, Riyadh 11586, Saudi Arabia; muaffaqnofal@gmail.com; 10 Centre for Foundation Studies in Science, University of Malaya, Kuala Lumpur 50603, Malaysia; mfzkadir@um.edu.my

**Keywords:** polymer blends, XRD and impedance study, TNM and LSV, CV, energy storage EDLC device

## Abstract

In this study, structural characterization, electrical properties and energy storage performance of plasticized polymer electrolytes based on polyvinyl alcohol/methylcellulose/ammonium thiocyanate (PVA/MC-NH_4_SCN) were carried out. An X-ray diffraction (XRD) study displayed that the plasticized electrolyte system with the uppermost value of direct current (DC) ionic conductivity was the most amorphous system. The electrolyte in the present work realized an ionic conductivity of 2.903 × 10^−3^ Scm^−1^ at room temperature. The main charge carrier in the electrolyte was found to be the ions with the ionic transference number (*t_ion_*) of 0.912, compared to only 0.088 for the electronic transference number (*t_elec_*). The electrochemical stability potential window of the electrolyte is 2.1 V. The specific capacitance was found to reduce from 102.88 F/g to 28.58 F/g as the scan rate increased in cyclic voltammetry (CV) analysis. The fabricated electrochemical double layer capacitor (EDLC) was stable up to 200 cycles with high efficiency. The specific capacitance obtained for the EDLC by using charge–discharge analysis was 132.7 F/g at the first cycle, which is slightly higher compared to the CV plot. The equivalent series resistance (ESR) increased from 58 to 171 Ω throughout the cycles, which indicates a good electrolyte/electrode contact. Ions in the electrolyte were considered to have almost the same amount of energy during the conduction process as the energy density is approximately at 14.0 Wh/kg throughout the 200 cycles. The power density is stabilized at the range of 1444.3 to 467.6 W/kg as the EDLC completed the cycles.

## 1. Introduction

The first solid polymer electrolyte (SPE) was made for the application in lithium batteries in 1979 and it is now one of the most important elements for applications to electrochemical devices [1]. Proton batteries and electrochemical double layer capacitors (EDLCs) are among the applications that are using SPEs and have been internationally recognized as an option for conventional batteries. While liquid electrolyte shows a good performance in a few energy devices, it can be easily evaporated and also damage the device’s internal elements due to corrosive and leakage problems [2,3,4]. Researchers therefore find that SPEs are the best candidate for replacing the liquid electrolytes considering their protection, long shelf life and also their easy manufacturing process [5]. Moreover, polymers used for polymer electrolyte preparation should have functional groups such as OH, C=O or NH_2_ on their backbone chains [6,7,8,9]. Besides, researchers find that EDLC has higher endurance and power density compared to other supercapacitors [10,11,12]. EDLC is produced through a simple and cost-effective fabrication process, mainly employing activated carbon, because of its good chemical stability and high electronic conductivity as well as its larger surface area compared to other fabrication materials [13,14,15]. Moreover, the energy storage of EDLC is manifested through a non-Faradaic mechanism because the ions develop a double layer by the interfacial area [16]. This explains that there is no electron transfer between the electrode surfaces but only charges will accumulate.

Natural polymers are broadly used in the preparation of polymer electrolytes due to their biodegradable properties that meet environmental and global energy requirements. Poly(vinyl alcohol) (PVA) and methylcellulose (MC) are examples of natural polymers that are widely researched in applications regarding electrochemical devices [17,18,19,20,21,22]. PVA is a non-toxic polymer that, when enriched with polar oxygen atoms from the vinyl alcohol groups, helps to produce complex salt cations, hence forming excellent polymer electrolyte complexes [23,24]. It has been shown to exhibit good characteristics such as high dielectric power, high chemical stability and good storage capacity [25]. It was found in the literature that PVA has shown good results as a host polymer both in terms of conductivity and electrochemical stability [26,27,28]. In addition, MC-based polymer electrolytes have recently been reported to have a high potential in the fabrication of EDLC [29]. MC is also stated to have good film forming characteristics, be biocompatible and low-cost as well as having good mechanical and electrical characteristics compared to other green polymers [30,31]. It is proposed that MC improves ionic conductivity and is commonly used in many different industries, including mineral processing, textile and medical applications [32]. Instead of using a single polymer to produce SPEs, the blending of two or more different polymers can improve mechanical strength, thermal stability and the availability of ion hopping sites [33,34].

Furthermore, salt addition has been introduced to the electrolyte to enhance its conductivity, which is a key parameter for the performance of the electrochemical devices. Although lithium-based salts show an overall good performance, their non-biodegradable characteristics may cause environmental pollution [35]. H^+^, Mg^2+^, and NH^4+^ are therefore alternatives to Li^+^ as cation providers in SPEs [2,36]. Hemalatha et al. [37] reported that the lattice energy of NH_4_SCN (605 kJ/mol) can cause the cation and anion to be easily dissociated during dissolution in a solvent. This phenomenon has led to an increase in ammonium ions in the polymer matrix provided by the NH_4_SCN [38]. The incorporation of NH_4_SCN salt reported an increment of room temperature conductivity for the carboxymethyl cellulose (CMC)-based polymer electrolyte up to 6.48 × 10^−5^ S/cm [39]. Besides, Premalatha et al. [40] have also reported that the blend of polyvinyl alcohol (PVA) and polyacrylic nitrile (PAN) films have achieved the maximum conductivity of 3.25 × 10^−3^ S/cm at room temperature with the addition of NH_4_SCN salt. The future challenge to achieve environmental friendly energy storage devices and minimize pollution have attracted the attention of many research groups. In fact, it is not an easy task to obtain the balance between good device performances, cost reduction and the use of eco-friendly materials. In this work we focused on the preparation and study of eco-friendly polymer electrolytes based on biodegradable polymers such as PVA and MC in an attempt to reduce electronic wastes. This work is a part of large effort and numerous researches carried out across the globe by scientists toward commercializing the biodegradable polymer electrolyte in energy devices such as batteries, supercapacitors and solar cells. In addition, from the economical view point, these types of biodegradable polymer electrolytes can play a main role in reducing the cost of electronic devices, both in terms of used raw materials and fabrication processes. The current research investigating the possibility and suitability of using biodegradable polymers in energy devices, which conceptually is a good realistic step toward the development and commercialization of eco-friendly energy devices. In the present work, the highest conducting electrolyte (PVA:MC blend with 40 wt.% NH_4_SCN and 48 wt.% Glycerol) have been studied using various experimental techniques and have been fabricated into the EDLC.

## 2. Experimental

### 2.1. Material and Preparation of Blend SPE Films

MC and PVA with the average molecular weight of 35,000 g/mol, obtained from Sigma Aldrich were used as raw materials in a powder form. NH_4_SCN salt has been used as an H^+^ ion provider for the polymer blend electrolyte. Raw materials were used to prepare PVA:MC:NH_4_SCN polymer blend electrolyte films through solution casting technique. The PVA:MC blends in 90:10 and 80:20 in weight percent were prepared. For this work, MC (10 and 20 wt.%) was dissolved separately in distilled water at an ambient temperature for 5 h. Concurrently, PVA (90 and 80 wt.%) were dissolved separately in distilled water (20 mL) at 80 °C. The PVA solution was allowed to cool down to room temperature. Then PVA and MC solutions were mixed under magnetic stirrer with continuous stirring. Consequently, for the PVA:MC (80:20) blend, 40 wt.% of NH_4_SCN salt dopant was added and stirred until a clear solution was obtained. Then, 14, 28, 42 wt.% of glycerol plasticizer (GCP) was added to the PVA:MC:NH_4_SCN electrolyte in order to prepare a plasticized polymer electrolyte and then the plasticized solutions were coded as PMNG-1, PMNG-2, and PMNG-3, respectively. Lastly, the solution mixtures were poured into different clean Petri dishes and left to evaporate gradually at an ambient temperature for several days.

### 2.2. EIS Measurements

Electrical impedance spectroscopy (EIS) is employed to analyze conductivity and other characteristics by measuring the impedance of electrolytes. At a frequency range of 50 Hz to 1000 kHz, HIOKI 3532-50 LCR HiTESTER is used for the measurement. Prior to this test, two stainless steel electrodes were used to place the selected electrolyte in between them before the measurement is taken. By using the following equation, room temperature conductivity can be calculated for each electrolyte [14,15].

(1) $$\sigma_{dc}=(\frac{1}{R_{b}})\times (\frac{t}{A})$$

where *t* and *A* represent the thickness of the electrolyte and the electrode–electrolyte contact area, respectively, while, *R_b_* is the bulk resistance which is taken from the spike–real axis interception.

### 2.3. LSV and TNM Studies

The decomposition voltage of the electrolytes exhibits their stability electrochemically, which can be evaluated through a linear sweep voltammetry (LSV) study. The LSV recording was carried out by using Digi-IVY DY2300 potentiostat at scan rate of 10 mV/s. Besides, transference number measurement (TNM) was computed through a V&A Instrument DP3003 at room temperature with a 0.2 V working voltage and a digital DC power supply. The electronic (*t_elec_*) and ionic (*t_ion_*) transference numbers were analyzed from the cell polarization versus time. The transference numbers for both ionic and electronic are calculated using the following equations [14,15]:

(2) $$t_{ion}=\frac{I_{i}-I_{ss}}{I_{i}}$$

(3) $$t_{elec}=\frac{I_{ss}}{I_{i}}$$

where *I_i_* and *I_ss_* are the initial current and steady state currents, respectively.

### 2.4. EDLC Preparation

**Scheme 1:**
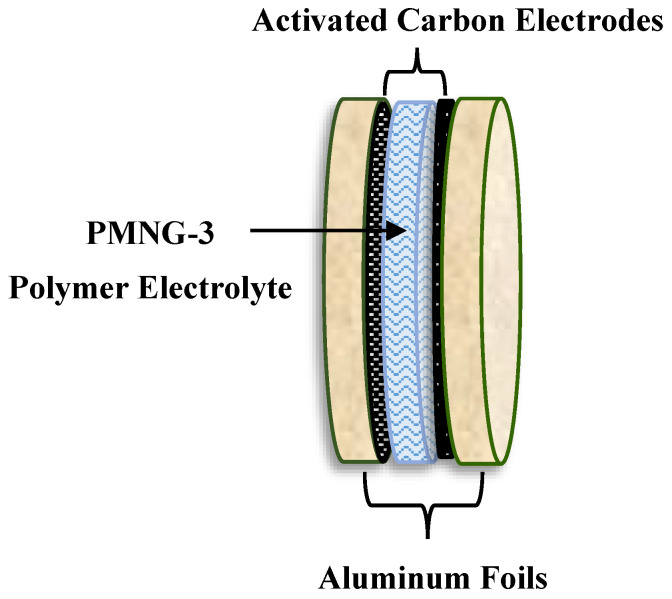
Schematic arrangement of the fabricated EDLC.

For the EDLC preparation in this study, the electrode was mainly fabricated using three main materials, which are carbon black, activated carbon and polyvinylidene fluoride (PVdF). Details of the electrode preparation were reported in our previous work [41]. Once the electrode was successfully prepared, the arrangement of the EDLC in a coin cell was (electrode | highest conducting electrolyte | electrode) as schematically illustrated in Scheme 1, and it is ready to be analyzed.

The performance of EDLC was first tested using a cyclic voltammetry (CV) analysis, where Digi-IVY DY2300 Potentiostat was occupied with a voltage range 0.0 to 0.9 V and sweep rates range of 10 to 100 mV/s. The specific capacitance (*C_s_*) from CV analysis can be calculated by using the equation below [21,22]:

(4) $$C_{cv}=\int_{V_{l}}^{V_{2}}\frac{I\left(V\right)dV}{2mv\left(V_{2}-V_{1}\right)}$$

where *I(V)dV* represents the area of CV curves, *m* is the active materials mass and *v* is the scan rate used during the measurements. *V*_1_ and *V*_2_ are 0.0 and 0.9 V, respectively.

Another important analysis of the prepared EDLC is the charge–discharge profiles, which was measured at a current density of 0.5 mA/cm^2^ by using a Neware battery cycler. By using the Equations (5) and (6), *C_s_* values from charge–discharge profiles as well as the equivalent series resistance (*ESR*) can be determined for 200 cycles [20,21,22].

(5) $$C_{s}=\frac{i}{ms}$$

(6) $$ESR=\frac{V_{drop}}{i_{}}$$

where *I* and *s* represent the applied current and the discharge slope, respectively while *V_drop_* is the voltage drop.

## 3. Results and Discussion

### 3.1. Structural Study

**Figure 1:**
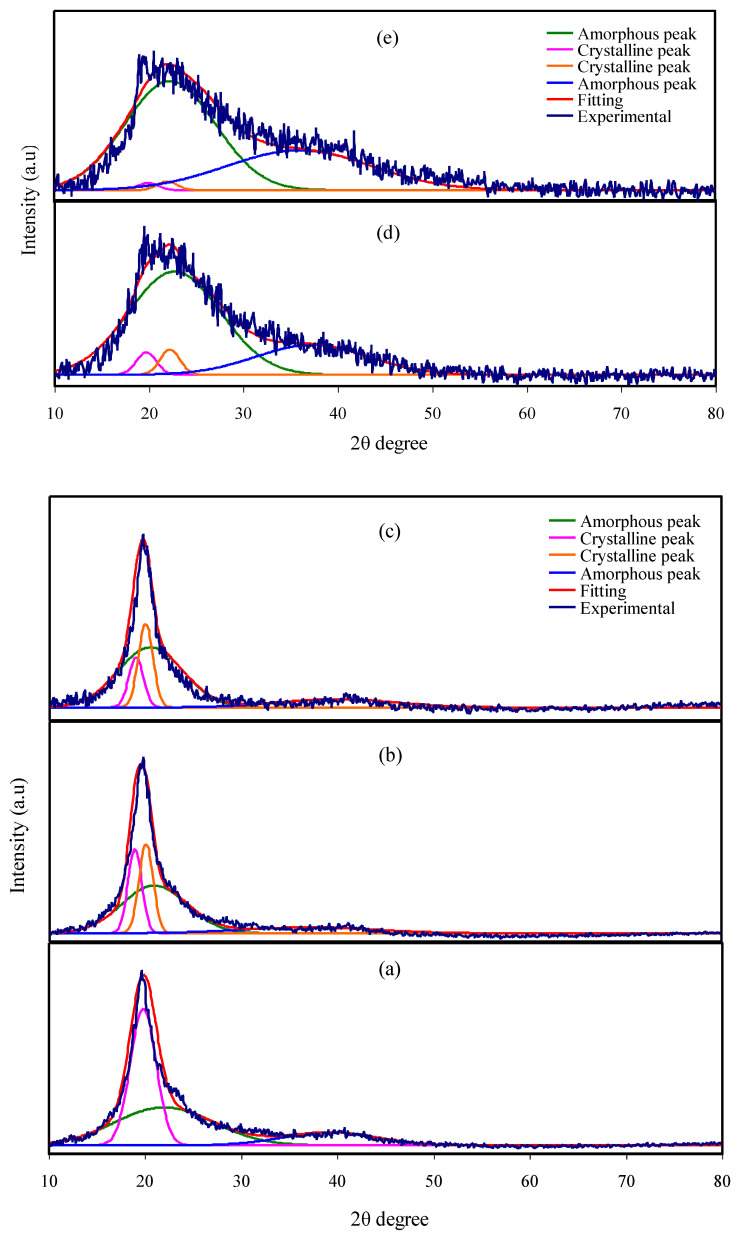
XRD spectra for (**a**) pure PVA, (**b**) 90 wt.% PVA:10 wt.% MC, (**c**) 80 wt.% PVA:20 wt.% MC, (**d**) PMNG-1, and (**e**) PMNG-3 electrolyte films.

Figure 1 shows the XRD patterns for the pure PVA, PVA:MC blends, and plasticized electrolyte films. It is documented that MC polymer possesses a unique peak at 2θ between 19° and 21°, arising from the inter-molecular hydrogen bonding coupled with a short distance order in the MC chains [42,43,44,45]. Early studies showed that the XRD spectrum of pure PVA possesses various crystalline peaks at 2θ = 20°, 23.43°, and 41.15° which are attributed to (101), (200), and (111) planes, respectively [46,47]. The emerged intense peak at 2θ = 18.6° in the XRD spectrum of pure PVA showed that the PVA has a semicrystalline structure [48]. The OH group existence along the PVA main chain is sufficient to offer strong intra-molecular and inter-molecular hydrogen bonding in PVA. This study has shown that as the concentration of MC increased from 10 to 20 wt.%, the intensity of the crystalline peaks of PVA decreased and the peaks underwent broadening (see Figure 1b,c). This is caused by disruption of hydrogen bonding due to the dominance of the amorphous phase in the blend system. Thus, polymer blending might be considered as an effective methodology to decrease the PVA crystalline segment. To determine the degree of crystallinity (*X_c_*), it is essential to deconvolute the XRD spectra of the samples to find the area of the amorphous and crystalline peaks [49]. The *X_c_* was calculated using Equation (7) [50].

(7) $$Xc=\frac{A_{C}}{A_{T}}\times 100\%$$

where *A_C_* and *A_T_* are the area of crystalline peaks and total area of amorphous and crystalline peaks, respectively. It is noteworthy to observe that the *X_c_* is decreased upon further increasing the MC content (see Table 1). The deconvoluted XRD spectra of the plasticized PVA:MC electrolyte is shown in Figure 1d,e. It is fascinating to observe that the intensity of the XRD peaks is considerably decreased upon an increasing in GCP. A previous study reported that salt inclusion into the polymer blends markedly reduced the *Xc* [20,21,51]. On the basis of previous research, salt can destroy the hydrogen bonding amongst the polymer chains because of electrostatic reactions created among the salt cations and polymer functional groups [52]. The amorphous structure increment might be associated with the crystalline phase disruption in the polymer [53]. Compared to the pure blend systems, the degree of crystallinity in the plasticized systems is very significantly diminished (see Table 1). Using plasticizers are famous approaches to raise the free ions number and decrease the *Xc* [54].

### 3.2. Impedance Analysis

**Figure 2:**
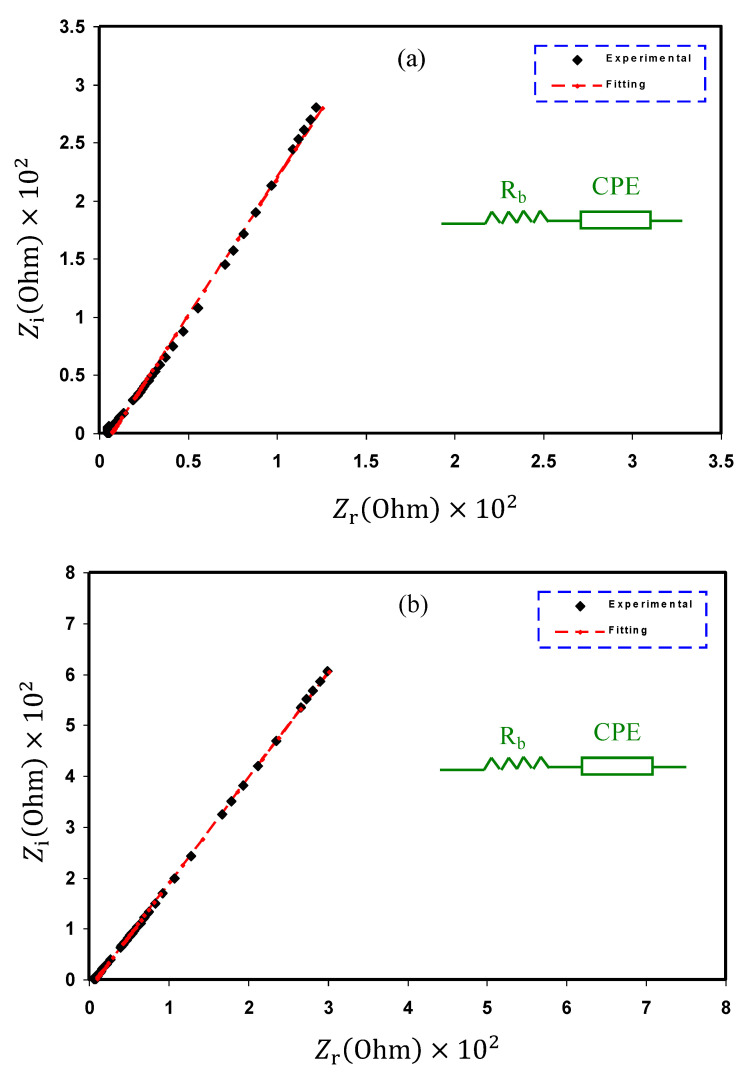

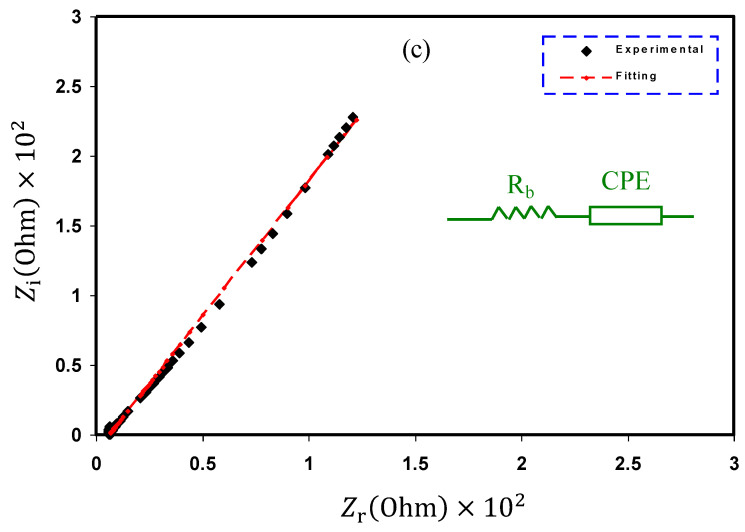
EIS plots for (**a**) PMNG-1, (**b**) PMNG-2, and (**c**) PMNG-3 electrolyte films.

Impedance spectroscopy is an effective method to investigate the electrical properties as well as to explore the conductive properties of polymeric materials that will be applied in electrochemical devices [6,55]. Over the years, the focus has been given to the ionic conducting materials because of the wide application range in solid electrochemical device fields [56]. The impedance plots at room temperature for the PVA:MC:NH_4_SCN:Gly electrolyte films are displayed in Figure 2a–c, where ($Z_{r}$) is the real part and ($Z_{i}$) is the imaginary part of the complex impedance ($Z^{*}$).

Based on Figure 2, a less than 90° inclined spike is detected at low frequency, which verifies the capacitive aspect in the electrolyte [57]. This situation illuminates the electrode polarization effect within the electrolytes [58,59]. The development of the electric double layer and free charge buildup are the cause for this phenomenon to happen at the electrolyte and electrode surface interface [60]. It is noticeable in Figure 2a,b that with rising GCP content the bulk resistance (R_b_) increased. This might be due to the association of cations and anions in the salt, which hinders ion transportation and segmental mobility, and thus causes the decrement in conductivity [61]. It is seen in Figure 2c that the R_b_ value is decreased as the GCP increases to 42 wt.% because of the charge carrier mobility increment, resulting in the increment in the conductivity [62].

The values of R_b_ are measured at the point where the semicircle intercepts the real axis (Z_r_). Equation (1) is employed to measure the films DC conductivity on the basis of R_b_ values and the films dimensions. Table 2 lists the DC conductivity for the plasticized system. The highest conductivity of the plasticized electrolyte system is a promise for application in EDLC. According to Kadir et al. [29], the addition of 30 wt.% GCP in MC:NH_4_Br electrolyte system showed the ionic conductivity of (1.67 ± 0.04) × 10^−3^ Scm^−1^. The conductivity value achieved by the electrolyte in this work is comparable to the literature that reported high conductivity such as 3.73 × 10^−3^ Scm^−1^ for lignin-based gel polymer electrolytes [35], 3.04 × 10^−4^ Scm^−1^ for corn starch/chitosan/ammonium iodide-based electrolytes [36] and 1.17 × 10^−3^ Scm^−1^ for PVA/amino acid proline/NH_4_SCN-based electrolytes [37].

The low frequency region would be presented by a constant phase element (CPE), which is related to the formed double-layer capacitance between the electrode and the blend electrolyte. The CPE is usually used in an electric equivalent circuit (EEC) rather than an ideal capacitor in real systems. This is because the real SPE behavior is unlike that of an ideal capacitor engaged in a pure semicircular pattern [63]. The spike character of the plasticized electrolytes indicates that just the resistive part of the polymer dominates [64]. In this case, the values of *Zr* and *Zi* associated with the EEC can be expressed as [22]:

(8) $$Z_{r}=\;R_{b}+\frac{A1}{C\;\omega^{p}}$$

(9) $$Z_{i}=\frac{A2}{C\omega^{p}}$$

where

$$A1=\cos\left(\frac{\pi p}{2}\right)\;\mathrm{and}\;A2=\sin\left(\frac{\pi p_{}}{2}\right)$$

where *C* is the CPE capacitance, *ω* refers to the angular frequency and *P* is associated with the vertical axis deviation of the impedance plots. The EEC fitting parameters are shown in Table 3.

### 3.3. TNM Analysis

**Figure 3:**
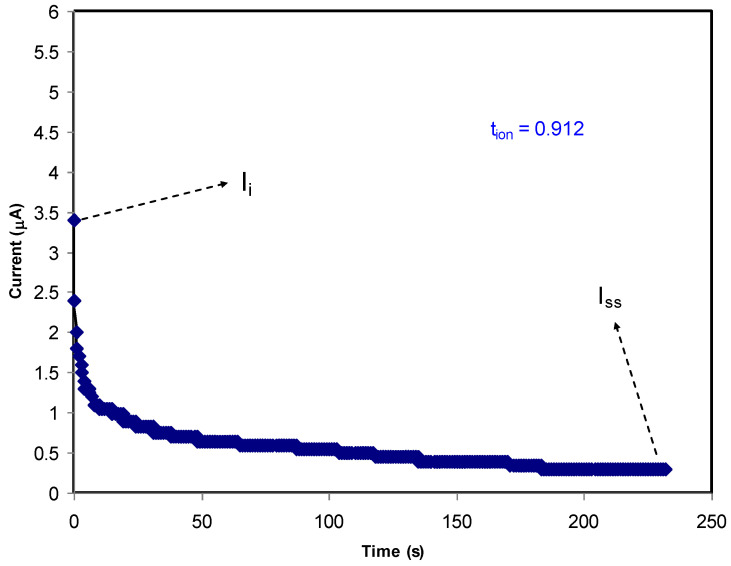
Current versus time for the PMNG-3 system.

The transference number measurement (TNM) analysis exhibits which charge carrier species is dominant within the electrolytes. When the ionic transference number (*t_ion_*) is near to unity, the system is confirmed to be dominated by ions [65]. The current plot against time for the PMNG-3 system is presented in Figure 3.

Based on Figure 3, the initial current is noticed to decrease as the time increases due to the depletion of ionic species, and a balanced mobile ion diffusion process will result in a steady state of current [66,67]. The ions are blocked by the stainless steel electrodes during the polarization process, while only electrons can flow through [68]. The electrolyte shows a *t_ion_* value of 0.912, whereas the *t_elec_* is 0.088 and this verifies that ions are the major carrier species in the PMNG-3 system. The result obtained for the PMNG-3 electrolyte is recommended for the good performance in the electrochemical devices application, particularly EDLC. This is due to the good DC conductivity value, which is recorded for this system. In addition to its sufficient electrochemical stability, which will be discussed in a later section. The glycerolized chitosan-NH_4_Br system also reported a comparable result [69].

### 3.4. LSV Study

**Figure 4:**
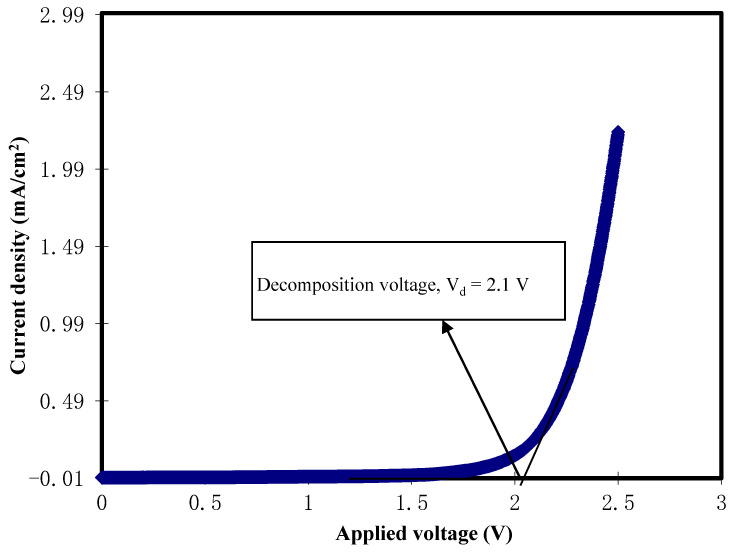
Linear sweep voltammetry (LSV) curve for the PMNG-3 system.

The linear sweep voltammetry (LSV) is used to examine the voltage that an electrolyte can operate without breakdown at room temperature [70]. Figure 4 illustrates the LSV plot of the PMNG-3 electrolyte at 10 mV/s. No current flow is observed in the electrolyte when the voltage is below 2.1 V, which explains that the electrochemical reaction does not occur within the electrolyte [71]. Therefore, the decomposition of the PMNG-3 system is observed to start at a voltage of 2.1 V, which is suitable enough to be used for the fabrication of EDLC that normally works at 1.0 V [72].

### 3.5. CV and EDLC Characterization

**Figure 5:**
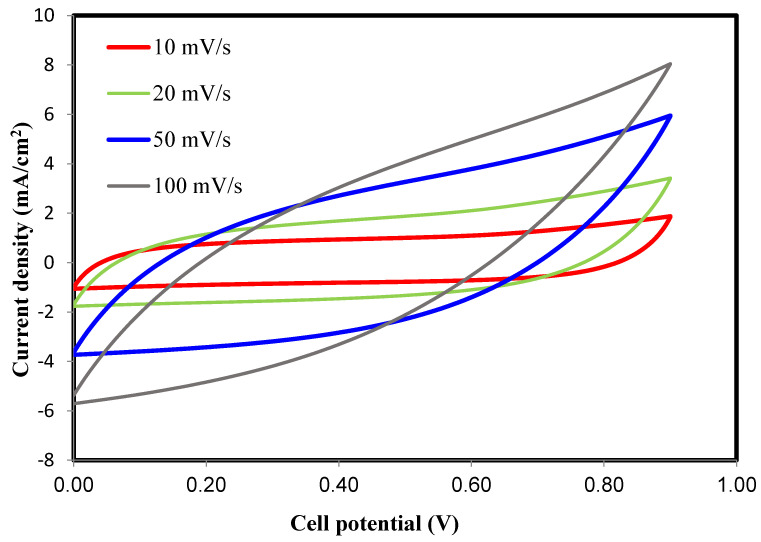
CV curve of the fabricated EDLC using PMNG-3 system.

The CV analysis is conducted to examine the behavior of the charge storage mechanism at the electrode–electrolyte interfaces of the fabricated EDLC. At different scan rates, the CV curve is recorded for the PMNG-3 electrolyte as shown in Figure 5.

When the scan rate increased, the curves are observed as changing from a rectangular shape to a leaf-shape, which is caused by the porosity of the carbon electrodes and internal resistance [73]. Based on the CV curves in Figure 5, no peak is observed, which means no oxidation/reduction process occurs in the EDLC using the PMNG-3 system. During the charging process of EDLC, the induced electric field at the positive electrode will attract the anions toward it from the electrolyte, while the negative electrode will attract the cations. The intense electric field from this phenomenon holds the electrons from electrodes and ions from the electrolyte [28]. This reveals that the double-layer charge is generated on the surface of carbon electrodes where the potential energy is stored [74]. The calculated *C_s_* values are listed in Table 4 at respective scan rates. Generally, a higher scan rate will cause the specific capacitance to decrease, which will reduce the stored charges on the electrode surface and the energy losses will get higher; thus, the *C_s_* values will decrease [75].

### 3.6. Charge–Discharge Study

**Figure 6:**
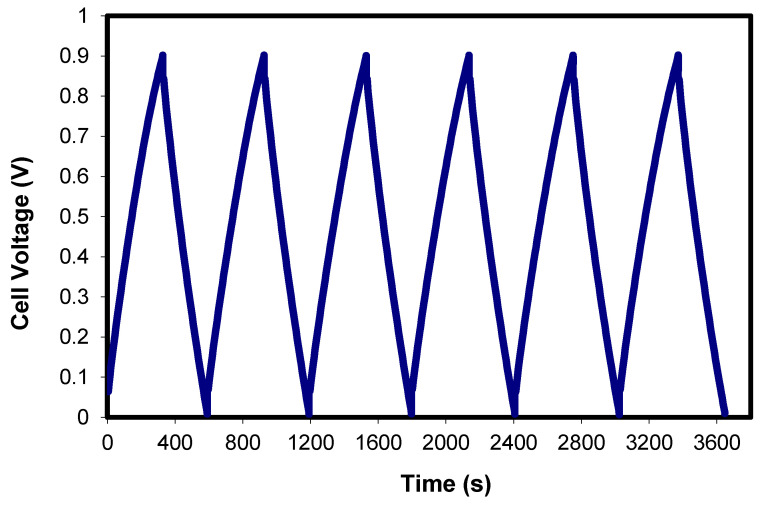
Charge–discharge profiles of EDLC at selected cycles.

The cyclic durability of the EDLC can be determined by using a charge–discharge profile [61]. The profile is used to depict the charging and discharging steps in the EDLC. The charge/discharge curves are shown in Figure 6 for selected cycles.

**Figure 7:**
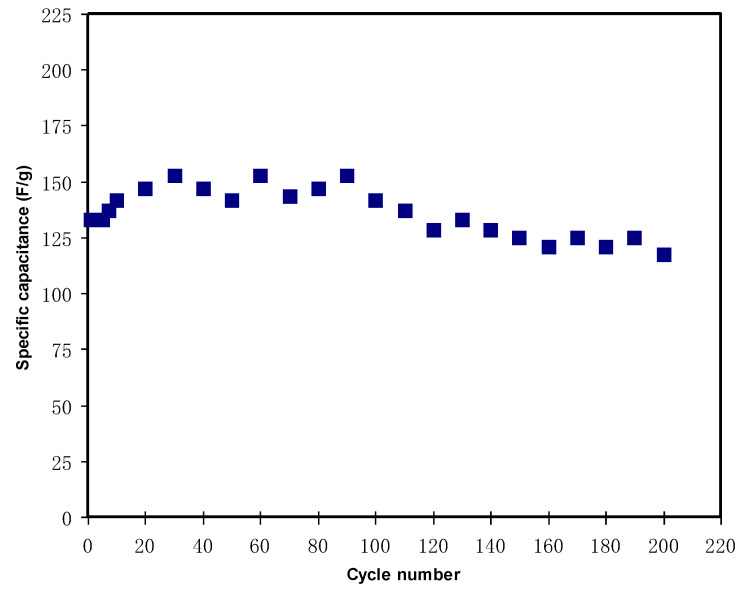
Specific capacitance of the EDLC for 200 cycles.

The capacitive behavior of the EDLC is confirmed when the discharge slope is almost linear as shown in the charge–discharge profile [19]. It is noticeable from Figure 7 that the calculated *C_s_* at the first cycle is obtained to be 132.7 F/g and is slightly higher than *C_s_* from CV curves. According to Yusof et al. [76], these differences are considered to be reliable since both methods prove the characteristics of the capacitor in the EDLC. Next, the *C_s_* is increased up to 152.4 F/g at the 90^th^ cycle and then gradually decreased to an average value of 124.7 F/g until the 200 cycles are completed. The downtrend of the *C_s_* value suggests the electrolyte depletion where ion pairs are possibly developed and contribute to the instability of the electrochemical devices [77]. The formation of ion pairs and ion aggregation caused the reduction in mobile charge carriers availability in the transportation between the electrodes. Hence, this will reduce the ion absorption onto the electrodes. A comparable *C_s_* value was reported by Muchakayala et al. [75] for the fabricated EDLC by using PVdF-HFP/[PMpyr][NTf_2_] with multiwalled carbon nanotube added activated carbon electrodes, which is at 156.64 F/g for 2000 cycles. Therefore, a new electrolyte material as shown in this work can be established in the EDLC with a promising specific capacitance. The authors declared that their electrolyte is a talented candidate for the development of flexible SCs. The specific capacitance values achieved in this work using CV and charge–discharge curves are compared with the previous works as displayed in Table 5.

**Figure 8:**
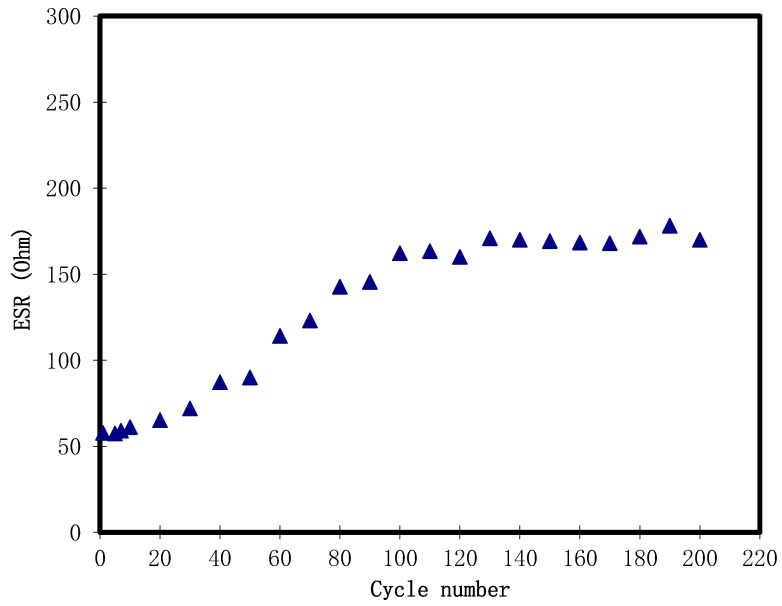
The plot of ESR of the fabricated EDLC for 200 cycles.

Prior to the discharging process beginning as in the charge–discharge profile (Figure 8), the tiny potential drops (*V_drop_*) could be due to the internal resistance in the EDLC that is also known as *ESR*, which can be calculated using Equation (6). Figure 8 displays the obtained *ESR* values for 200 cycles. Based on the *ESR* plot, it can be observed that at the 1st cycle *ESR* value is achieved to be 58 Ω and then rises to 171 Ω at the 130th cycle. The results are noticed to remain at a constant reading throughout the 200 cycles.

It is worth mentioning that the current proton conducting polymer electrolytes based on NH_4_SCN salt exhibits a good DC conductivity of 2.24 × 10^−3^ S cm^−1^. This value is promising for the energy device applications, and it outweighs many other non-proton conducting-based polymer electrolytes such as lithium perchlorate (LiClO_4_)-based electrolytes with a conductivity of 7.34 × 10^−4^ S cm^−1^ [51], magnesium chloride (MgCl_2_) with a conductivity of 1.03 × 10^−3^ S cm^−1^ [54], and 2.26 × 10^−4^, 4 × 10^−5^, and 1.5 × 10^−7^ S cm^−1^ for the lithium-based salts LiClO_4_, LiF_3_CSO_3_, and LiBF_4_, respectively [58]. Moreover, the satisfactory electrochemical stability of 2.1 V in the current work makes the fabricated polymer electrolytes a suitable candidate for the EDLC applications, and opens a gate toward commercializing proton conducting polymer electrolytes. However, in order to meet the industrial level and enhance the EDLC performance, further improvement in the conductivity of polymer electrolytes with establishing good electrode and electrolyte contact are highly demanded.

The small *ESR* value portrays a good electrode–electrolyte contact and also proves that there is a low resistance for the migration of ions to form electrical double-layer [81]. According to Arof et al. [82], there are a few factors that influenced the existence of the internal resistance; the charging–discharging process of electrolytes, current collector types which are aluminum foils, and the electrolyte–electrode gap. Aziz et al. [19] have reported a similar trend for *ESR* values in the chitosan/methylcellulose-NH_4_SCN system, where the *ESR* values are increased at the constant values of *C_s_*. The recombination of free ions will have occurred from the rapid charging and discharging process, which results in the development of an ion pair, hence the conductivity will decrease.

**Figure 9:**
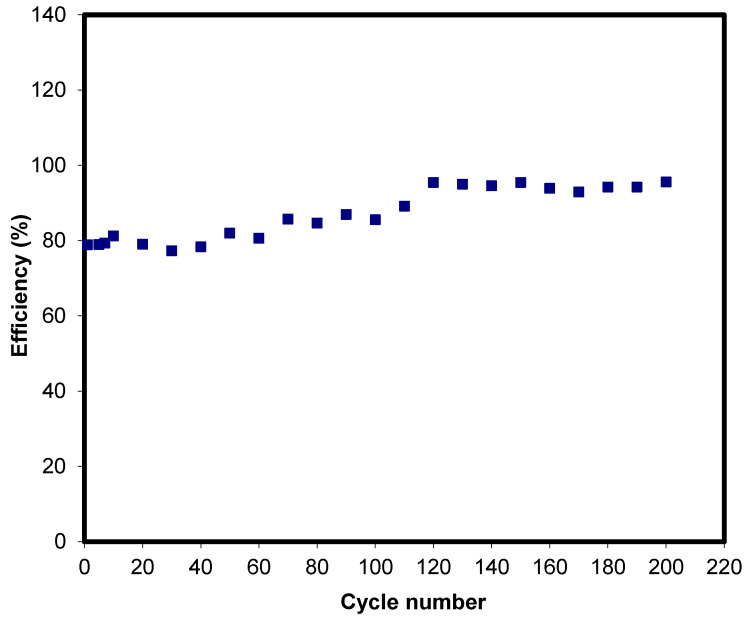
The plot of Coulombic efficiency (η) of the fabricated EDLC for 200 cycles.

It is important to determine the Coulombic efficiency (η) of the EDLC in order to study its performance and cycling stability. This parameter can be obtained by using Equation (10) [22] and is presented in Figure 9 for 200 cycles.

(10) $$\eta =\frac{t_{d}}{t_{c}}\times 100$$

where *t_c_* and *t_d_* are the charge and discharge times, respectively. The efficiency is found to be 78.9% at the 1st cycle and then slightly increases to 89.2% at 110th cycle. The efficiency is then observed to remain approximately 95.0% at the 120th cycle and onwards up to 200 cycles. According to Lim et al. [83], the fabricated EDLC is considered as a likely contact between electrodes and electrolytes when the efficiency is more than 90.0%.

Other parameters to describe the performance of EDLC are the energy density (*E*) and as well as the power density (*P*). These parameters are calculated by using Equations (11) and (12) [20,21,22]:

(11) $$E=\frac{1}{2}(C_{s}V^{2})$$

(12) $$P_{}=\frac{V^{2}}{4m(ESR)}$$

**Figure 10:**
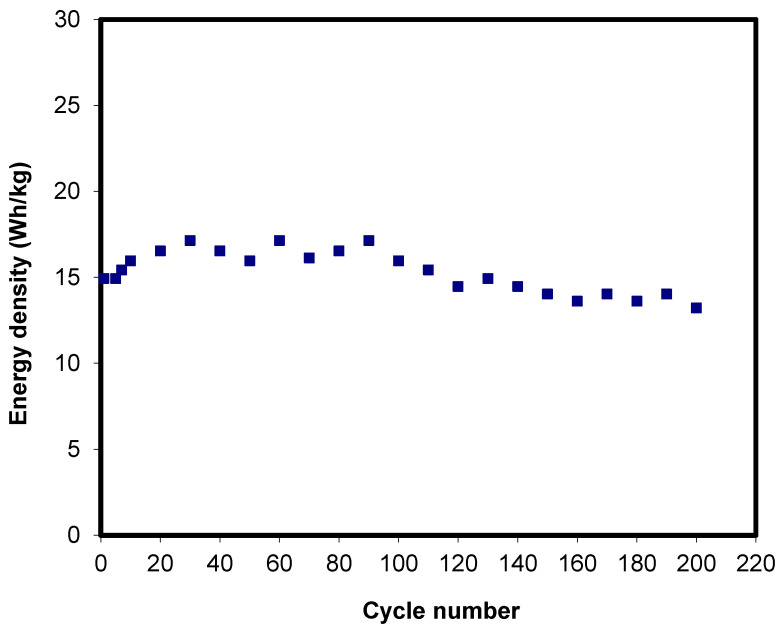
Energy density of the EDLC for 200 cycles.

**Figure 11:**
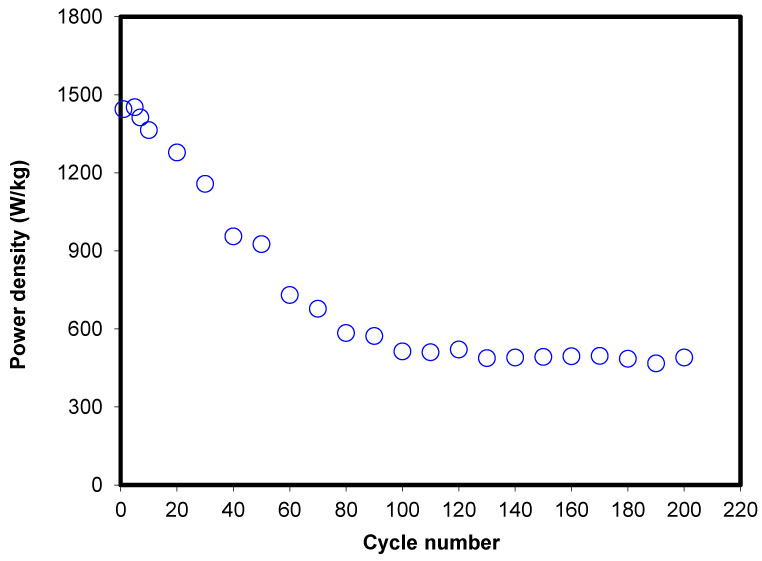
Power density of the EDLC for 200 cycles.

Figure 10 illustrates the energy density for 200 cycles. The energy density is found to be at 14.9 Wh/kg for the 1st cycle, and it is gradually increased to 17.1 Wh/kg at the 90th cycle. The energy density is constant at around 14.0 Wh/kg at the 100th cycle onwards until the 200th cycle. This trend is in good agreement with the pattern of *C_s_* showed in Figure 7. Shukur et al. [64] reported a comparable trend and the authors stated that the amount of energy required for the whole charging–discharging process is similar to the amount required for the migration of charge carriers towards the surface of the electrodes. Furthermore, the power density values are calculated and plotted in Figure 11. It can be observed that the *p* value at the 1st cycle is 1444.3 W/kg. The value is decreased to 572.7 W/kg at the 90^th^ cycle and then continuously dropped to 467.6 W/kg at the 200th cycle. Besides, the observation on the power density shows that the trend is complementary to Figure 8 for the *ESR* plot. The occurrence of electrolyte depletion is a result of the increment of internal resistance, hence the fast charging–discharging process will cause the ions to rapidly recombine and therefore the power density will reduce at a high cycle number [84].

## 4. Conclusions

Solid polymer electrolytes, which were hosted by a PVA/MC-NH_4_SCN polymer blend with a glycerol plasticizer, were successfully prepared by using the solution casting method. XRD technique was displayed in the plasticized electrolyte system with the maximum value of ionic conductivity being the most amorphous system. The ambient temperature DC conductivity of the highest plasticized SPE was found to be 2.903 × 10^−3^ S/cm. The TNM analysis confirmed that the ions were the dominant charge carriers in the electrolyte where *t_ion_* and *t_elec_* values were 0.912 and 0.088, respectively. The LSV analysis has shown that the electrochemical stability of the electrolyte is 2.1 V, which is high enough to be applied in EDLC. The CV analysis verified the capacitive characteristics of the EDLC since there were no observable peaks and when the scan rate is increased from 10 mV/s to 100 mV/s, the *C_s_* is reduced from 102.88 F/g to 28.58 F/g. The fabricated EDLC was also stable up to 200 cycles, with a steady efficiency at approximately 95.0%. The *C_s_* from the charge–discharge analysis was 132.7 F/g at the 1st cycle, which is slightly higher that *C_s_* from the CV plot. The *ESR* value was found to be 58 Ω and increased to 171 Ω throughout the cycles. The energy density is approximately constant at 14.0 Wh/kg, while the power density is stabilized at the range of 1444.3 to 467.6 W/kg as the EDLC completed the cycles.

## Figures and Tables

**Table 1:** The *X_c_* using deconvoluted XRD examination.

Electrolyte	Degree of Crystallinity (%)
Pure PVA	41.68
PVA:MC (90:10)	38.52
PVA:MC (80:20)	28.57
PMNG-1	6.88
PMNG-3	1.98

**Table 2:** DC conductivity for PVA:MC:NH_4_SCN:Gly systems at room temperature.

Designation	R_b_ (Ohm)	Conductivity (S cm^−1^)
PMNG-1	7	1.92 × 10^−3^
PMNG-2	8.5	1.58 × 10^−3^
PMNG-3	6	2.24 × 10^−3^

**Table 3:** The EEC fitting parameters for PVA:MC:NH_4_SCN:Gly systems at room temperature.

Sample	K (*F*^−1^)	C (*F*)
PMNG-1	2.2 × 10^4^	4.55 × 10^−5^
PMNG-2	4.05 × 10^4^	2.47 × 10^−5^
PMNG-3	1.40 × 10^4^	7.14 × 10^−5^

Where K is the reciprocal value of specific capacitance and it measured in F^−1^.

**Table 4:** Calculated *C_s_* of the EDLC from CV curves.

Scan Rate (mV/s)	*C_s_* (F/g)
10	102.88
20	85.28
50	52.58
100	28.58

**Table 5:** The specific capacitance values of EDLCs using various SPEs.

SPEs	Specific Capacitance (F/g) Using CV Curve	Specific Capacitance (F/g) Using Charge-Discharge Curve	Ref.
MC:NH_4_NO_3_:PEG	25 at 1 mV s^−1^	38	[78]
MC:PS:LiClO_4_:glycerol	61.58 at 2 mV s^−1^	28.04	[79]
PS:MC:NH_4_NO_3_:glycerol	20.48 at 1 mV s^−1^	31	[80]
CS:MgCl_2_: glycerol	50 at 10 mV s^−1^	117	[54]
CS:MC:NH_4_I:glycerol	9.97 at 100 mV s^−1^	9.7	[21]
CS: Mg(CH_3_COO)_2_: glycerol	39.72 at 10 mV s^−1^	78.2	[54]
**PVA:MC:NH_4_SCN:glycerol**	**102.88 at 10** **mV s^−1^**	**132.7**	**This work**
